# Coadministration of Compound Danshen dripping pills and bezafibrate has a protective effect against diabetic retinopathy

**DOI:** 10.3389/fphar.2022.1014991

**Published:** 2022-10-05

**Authors:** Le Liu, Xiaoqiang Li, Wenbin Cai, Kaimin Guo, Xuelian Shi, Lu Tan, Yao Zhan, Xueshuang Jing, Wenjia Wang, Shuiping Zhou, He Sun, Xu Zhang, Yunhui Hu

**Affiliations:** ^1^ Tianjin Key Laboratory of Metabolic Diseases, Collaborative Innovation Center of Tianjin for Medical Epigenetics, Center for Cardiovascular Diseases, Tianjin, China; ^2^ Research Center of Basic Medical Sciences, Department of Physiology and Pathophysiology, Tianjin Medical University, Tianjin, China; ^3^ Cloudphar Pharmaceuticals Co., Ltd., Shenzhen, China; ^4^ Department of Laboratory Animal Science and Technology, Tianjin Medical University, Tianjin, China; ^5^ Research Center of Basic Medical Sciences, Tianjin Medical University, Tianjin, China; ^6^ Tasly Pharmaceutical Group Co., Ltd., Tianjin, China

**Keywords:** diabetic retinopathy, compound Danshen dripping pills, bezafibrate, inflammation, oxidative stress

## Abstract

Diabetic retinopathy (DR) is increasingly becoming a main complication of diabetes, and is difficult to cure. In our research, network pharmacology analysis suggested that both compound Danshen dripping pills (CDDP) and bezafibrate (BZF) have potential protective effects against DR and the two drugs may act synergistically. The pharmacological effects of the coadministration of CDDP and BZF were elucidated in db/db mice, which simulate DR. Fluorescein fundus angiography showed that coadministration attenuated vascular leakage. Optical coherence tomography and hematoxylin and eosin staining showed that coadministration improved retinal thickness better than CDDP monotherapy. In addition, cell fluorescence images of reactive oxygen species revealed that coadministration of CDDP and BZF had more potent effects against oxidative stress than CDDP monotherapy. Metabolomics analysis showed that coadministration reduced the ratio of oxidized glutathione to reduced glutathione further than CDDP monotherapy. Coadministration of CDDP and BZF may provide additional protective effects by resisting vascular leakage, increasing retinal thickness, and inhibiting inflammation and oxidative stress in DR.

## Introduction

Diabetic retinopathy (DR) is the leading cause of visual impairment and blindness in diabetic patients. As the prevalence of diabetes continues to rise and patients live longer, the number of patients with DR is increasing. Depending on the degree of progression and the manifestation of retinopathy, DR can be divided into non-proliferative diabetic retinopathy (NPDR) and proliferative diabetic retinopathy (PDR). NPDR is the most common form of DR and manifests as leakage of fluid or blood from small blood vessels within the retina, which can develop into small bulges. The area of the retina affected by the leakage swells, resulting in partial visual field damage. With poor control, NPDR can develop into PDR, the treatment of which often requires anti-vascular endothelial growth factor (VEGF) drugs, total retinal laser photocoagulation, and sometimes vitrectomy. Optimal control of blood glucose and blood pressure in individuals with diabetes remains the cornerstone for preventing the development and arresting the progression of DR (Wong et al., 2016). Therefore, the development of strategies more specific to the pathophysiology is needed.

The pathophysiological mechanism related to DR includes inflammation, oxidative stress, microcirculation dysfunction, and retinal cell apoptosis. Compound Danshen dripping pills (CDDP) is a Chinese traditional medicine formula mainly used for the treatment and prevention of coronary heart disease. CDDP is composed of Danshen (Radix Salviae Miltiorrhizae), Sanqi (Radix Notoginseng), and borneol. Due to the vascular protective effects of the active ingredients (Hu et al., 2021), CDDP is considered as a potential therapeutic agent for DR. A randomized, double-blind, placebo-controlled multicenter clinical trial showed that CDDP was safe and effective for treating DR (Lian et al., 2015). A pharmacological mechanism study demonstrated that CDDP inhibited apoptosis in rat retinal cells (Zhang et al., 2018). However, it is not clear whether CDDP has other protective effects on animals with DR.

Diabetic patients often have dyslipidemia, which is also an important factor in DR progression (Hammer and Busik, 2017) and mainly manifests as hypertriglyceridemia. Clinical evidence suggests that fibrates can inhibit the progression from NPDR to PDR, but the mechanism is unknown (Meer et al., 2022).

In the present work, to predict the effect of the combination of bezafibrate (BZF) and CDDP, we performed a network pharmacology analysis to examine the proximity of the drug targets to the gene module of DR and whether they are topologically separated from each other in the same network neighborhood. We used db/db mice, which are a spontaneous diabetic model of type 2 diabetes, as the animal model of DR to examine the pharmacological effects of coadministration of CDDP and BZF experimentally. Coadministration of CDDP and BZF provided better protection for DR than CDDP alone. In the coadministration group, the effect on rescuing retinal thinning was stronger. Thus, BZF brings additional benefits, including the inhibition of retinal epithelial cell apoptosis and inhibition of inflammation and oxidative stress.

## Materials and methods

### Literature mining of targets for CDDP and BZF

Targets of CDDP were collected from related literature in the CNKI and PubMed databases using multiple keywords, including “CDDP,” “compound Danshen dripping pills,” and “compound Danshen dropping/drop pills.” A total of 140 genes (Supplementary Table S1) with Entrez ID were kept after careful manual examination. BZF targets were searched using the literature mining function of the LTM-TCM database (Li et al., 2022) (http://cloud.tasly.com/#/tcm/home, (Li et al., 2022). For BZF, 45 targets were supported by at least two publications. After manual examination, 25 targets associated with BZF were obtained (Supplementary Table S2).

### Collection and analysis of genes associated with DR

A total of 645 disease genes associated with DR were downloaded from DisGeNET Database *via* searching using the keyword ‘diabetic retinopathy’. After filtering, 276 genes with Score_gda >0.01 (Supplementary Table S3) were kept and were used for subsequent analysis.

Disease proteins are not scattered randomly in the interactome, but tend to form localized neighborhoods, known as disease modules (Menche et al., 2015). Therefore, a network approach, called the largest connected component (LCC) approach (Fang et al., 2021), was used to build a disease module for DR. In brief, we rebuilt a new network module by searching the LCC formed by the list of 276 of filtered DR-associated genes. We performed permutation analysis to evaluate the significance of the disease module as follows.

$$p-\text{value}=\frac{No.\left\{S_{m}\left(p\right)>S_{m}\right\}}{No.\left\{total\;permutations\right\}}$$ (1)

A nominal *p*-value was computed by counting the number of permutations (*S*_*m*_(p)) greater than the number of observed LCCs (*S*_*m*_) formed by the randomly selected proteins with a similar connectivity distribution in the human interactome network. We performed the permutations 1000 times to calculate the statistical significance.

The network graph of the interaction of genes related to the disease was visualized by using Cytoscape software (Shannon et al., 2003). Functional annotation of disease genes was conducted by using MetaCore database (https://portal.genego.com/).

### Correlation of drug targets and disease genes

The correlation of the drug targets and the genes associated with the disease *via* network propagation was calculated. Briefly, we took drug targets and disease genes as seed genes to run the random walk with restart algorithm (Kohler et al., 2008) in the human interactome network (Fang et al., 2021) as the background network. The influence score vector of the two sets of seed nodes on all nodes in the background network was obtained. The Pearson correlation coefficient of the two score vectors was then calculated, and the Z-score, calculated by performing permutations 1000 times, was used to evaluate the significance of the correlation. In the permutations, each of the 1000 groups of random contrast disease genes contained the same number of randomly selected proteins as the disease seed nodes.

### Network proximity analysis

The human interactome network was used as the background network in network proximity analysis. Network proximity between the drug (X) and disease (Y) was evaluated by the Z-score method (Cheng et al., 2019) (z = $\frac{d-\mu }{\delta }$ ), an efficient technique that relies on the shortest path lengths *d*(*x*, *y*) between drug targets (*x*) and disease proteins (*y*).

$$d\left(X,Y\right)=\frac{1}{\left|\left|X\right|\right|}\underset{x\in X}{\sum }\min_{y\in Y}d\left(x,y\right)$$ (2)

The Z-score is obtained by comparing the observed distance with a reference distance distribution between a randomly selected group of proteins of matching size and degree distribution as the disease proteins in the human interactome.

Proximity between the two drugs was calculated by the network proximity index proposed by Barabasi et al. (Menche et al., 2015). The network proximity of drug target modules A and B is defined using the separation measure (*S*_AB_),

$$S_{AB}={\langle d}_{AB}\rangle -\frac{\langle d_{AA}\rangle +\langle d_{BB}\rangle \;}{2}$$ (3)

which compares the mean shortest distance within the interactome of each target module, ⟨*d*_*AA*_⟩ and ⟨*d*_*BB*_⟩, to the mean shortest distance ⟨*d*_*AB*_⟩ between target modules A and B. If *S*_*AB*_ < 0, the targets of the two drugs are located in the same neighborhood, suggesting that they have similar effects. If *S*_*AB*_ ≥ 0, the targets are topologically separated, and thus the two drugs may have different effects.

### Animal experiments

Six-week-old db/db mice and BKS mice were purchased from China Jiangsu Jicui Yaokang Biotechnology Co., Ltd. All animal protocols complied with all relevant ethical regulations and were approved by the Animal Care and Use Committee of Tianjin Medical University. The mice in the control group (BKS mice) and the model group (db/db mice) were fed a standard diet, and the mice in each treatment group were administered CDDP (1600 mg/kg/d) or CDDP (1600 mg/kg/d) and BZF (75 mg/kg/d) for 16 weeks (*n* = 20). CDDP was prepared by Tasly Pharmaceutical Group Co., Ltd. (Tianjin, China). BZF was purchased from Shanghai Lanmu Chemical Co., Ltd.

### Fluorescein fundus angiography

The changes in retinal vascular permeability in mice were detected by fluorescein fundus angiography (FFA; MicroIV, Phoenix Research Labs). The mice were anesthetized with 1% tropicamide eye drops and their eyes were dilated. The eyeballs were covered with ofloxacin eye ointment. After injection of 2.5% sodium fluorescein (0.1 ml) for 2 min, fundus examination was performed and a digital fundus camera was used for photography.

### Optical coherence tomography

The retinal thickness of mice was measured by optical coherence tomography (OCT; MicroIV, Phoenix Research Labs). Mice were anesthetized and eyes were dilated with 1% tropicamide eye drops, and the eyeballs were covered with ofloxacin eye ointment. Images were collected and the retinal thickness was calculated.

### Hematoxylin and eosin staining

After euthanasia, the eyeballs of mice were quickly removed and fixed with eyeball fixative, embedded in paraffin, and sectioned. Paraffin sections of the eyes were stained with hematoxylin and eosin (H&E). Retinal thickness was measured after taking photographs.

### Measurement inflammatory cytokines

To evaluate the inflammatory cytokines, commercial assay kits were used in this study. Plasma was obtained by removing blood cells through centrifugation at 3,000 *g* for 10 min at room temperature. The concentrations of TNF-α, IL-18, IL-6, IL-1β, MCP-1, ICAM-1 and VEGF were measured using the ELISA kits (YITE Life-science, Tianjin, China) according to the manufacturer’s instructions.

### Cell culture and treatment

To establish a hyperglycemia model *in vitro*, human retinal pigment epithelium cells (ARPE-19 cells) were cultured in DMEM/F12 (DMEM/F12, Gibco, Grand Island, NY, United States) medium supplemented with 10% fetal bovine serum (FBS), and treated with high glucose (50 mM) for 72 h under the standard cultured conditions (37°C, 5% CO_2_). CDDP monotherapy and coadministration with BZF were performed with APRE-19 hyperglycemia cells, and solvent treatment served as the control group.

### TUNEL staining

Cell apoptosis were detected by using a TUNEL Apoptosis Detection Kit (Orange Fluorescence) (Abbkine Scientific Co., Ltd., Wuhan, China). After antigen recovery, sections were infiltrated with 0.1% Triton X-100 for 10–30 min, and then incubated overnight with the reaction mixture at 4°C. The next day, the sections were stained with DAPI, and then observed and recorded with fluorescence microscope.

### Cell reactive oxygen species assay

Intracellular reactive oxygen species (ROS) levels were measured using a ROS kit. ARPE-19 cells were washed twice with phosphate-buffered saline and incubated with 10 μM cell-permeable DCFH-DA for 20 min. Subsequently, the cells were washed with culture medium without fetal bovine serum, and their fluorescence was measured quantitatively by confocal laser scanning microscope with excitation and emission wavelengths of 488 and 525 nm, respectively.

### Western blotting

After 72 h of treatment with drugs and high glucose concentration, the cells were collected with RIPA lysis buffer. The extracted protein was separated by SDS-PAGE, and then transferred to the nitrocellulose membrane. After blocking for 1 h with 5% milk, the membrane was incubated with the primary antibody overnight, and then cultured with the secondary antibody at room temperature. Enhanced chemiluminescence was measured and photographs were taken.

### Profiling of redox-related metabolites

The cell samples were separated on an ultra-performance liquid chromatography BEH T3 column (Waters; 1.7 μm, 100 × 2.1 mm i.d.) at a flow rate of 0.3 ml/min. The column was maintained at 25°C, the sample chamber was at 4°C, and the injection volume was set to 10 μL. The polar compound mobile phases were water containing 0.25 mM di-*n*-butylamine acetate (solution A) and acetonitrile containing 3 mM di-*n*-butylamine acetate (solution B). Targeted profiling of metabolites was performed with a hybrid triple quadrupole linear ion trap mass spectrometer (5500 QTRAP, AB SCIEX) equipped with a turbo ion spray electrospray ionization source operating in negative mode.

## Results

### Network pharmacology analysis of CDDP and BZF for DR

**FIGURE 1:**
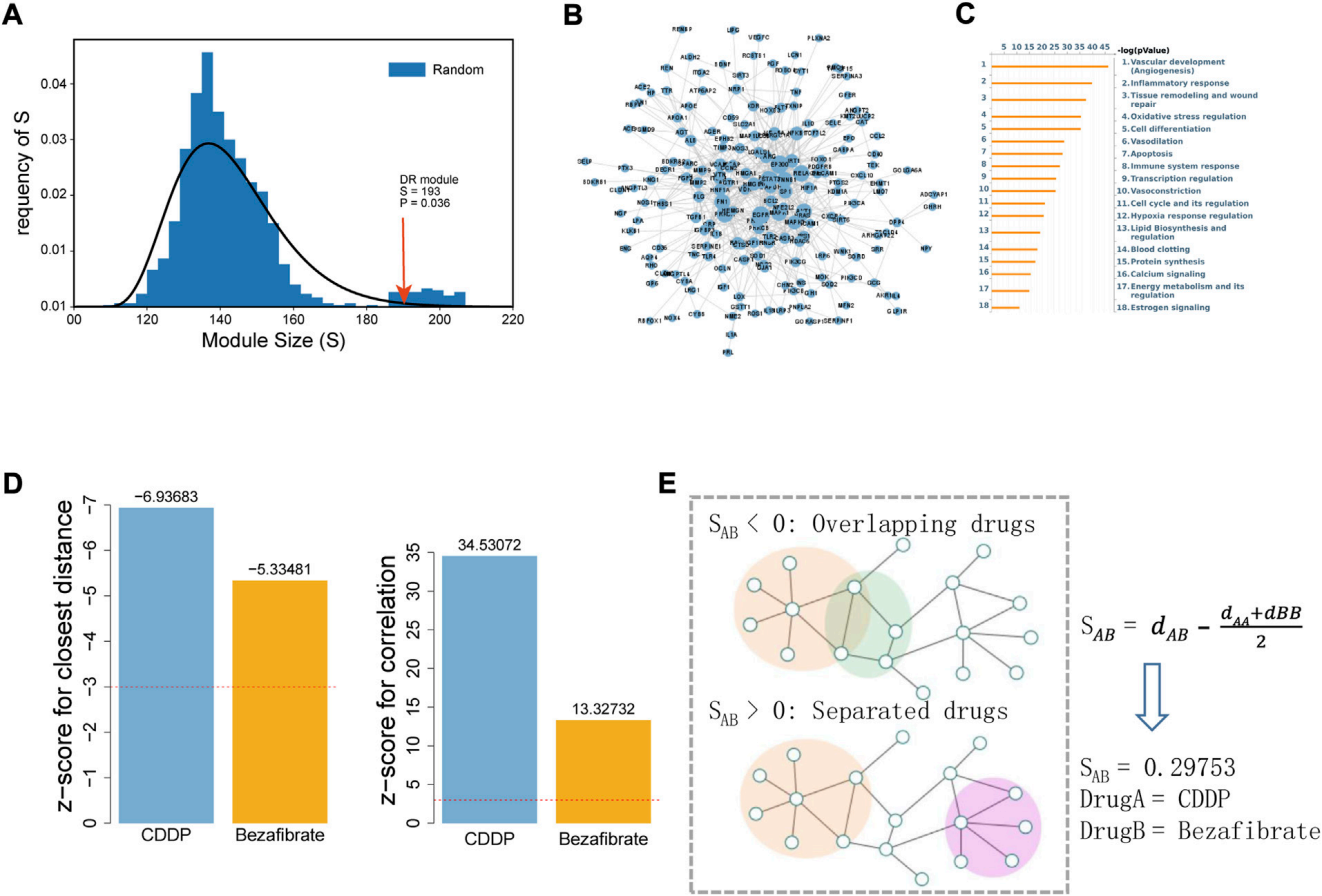
Correlation analysis between DR and the drugs (CDDP and BZF) based on the human interactome network. **(A)** LCC analysis of DR-associated genes. Size of the LCC module of DR is shown by the arrow (*p*-value was calculated with 1000 permutations). **(B)** Interaction network of LCC genes for DR visualized by Cytoscape. **(C)** Enrichment analysis of LCC by the MetaCore database. The 18 most significant biological processes are shown sorted by *p*-value. **(D)** Efficacy of CDDP and BZF on DR evaluated by network proximity (left) and correlation (right). The Z-score was used to evaluate their significance, and a Z-score of <3 (for closest distance) or >3 (for correlation) was considered to be significant. **(E)** Network proximity analysis of drug target modules A and **(B)**. *S*_*AB*_ was used to evaluate the network proximity between target modules A and **(B)**. *d*_AA_ and *d*_BB_ are the shortest distances within the interactome for target modules A and B, respectively, and *d*_AB_ is the shortest distance between target modules A and **(B)**.

DR-associated genes were collected from the DisGeNET database, and 276 genes were obtained after filtration based on the confidence score (Score_gda >0.01). Disease genes tend to be clustered in the background network to form a disease module *via* interacting with each other (Menche et al., 2015). LCC analysis was conducted using DR-associated genes. Of the 276 genes, 249 were in the background network and 193 were significantly connected (compared with random sampling, *p* = 0.036), forming a connected sub-network (Figures 1A,B). Functional annotation of 193 genes was conducted using MetaCore database (Figure 1C), and the significantly enriched pathways included angiogenesis, inflammatory response, oxidative stress, and apoptosis, indicating the reliability of DR-associated genes and providing a guarantee that the correlation between the drugs and disease was calculated accurately.

Subsequently, the correlation between the drug targets (CDDP and BZF) and the DR-associated genes was calculated by network proximity and correlation. The network proximity calculations between CDDP and BZF and the DR-associated genes were significant, with Z-scores of −6.94 and −5.33, respectively (Figure 1D, left). The targets of CDDP and BZF were positively correlated with DR-associated genes on the network, and the correlations were significant with Z-scores of 34.53 and 13.73, respectively (Figure 1D, right). These results indicated that both CDDP and BZF had potential efficacy for treating DR.

To investigate whether CDDP combined with BZF had a better effect for treating DR, the network proximity index proposed by Barabasi et al. was used to explore the interaction between the two drugs (Figure 1E). In this method, *S*_AB_ is used to represent the proximity on the network for drugs A and B. The network proximity of the targets between CDDP and BZF was 0.29753 (Figure 1E), showing that the targets of CDDP and BZF are topologically separated in the background network, and thus have different mechanisms of action on the disease. In summary, the combination of CDDP and BZF was promising for treating DR.

### Coadministration of CDDP and BZF significantly attenuated the DR disease phenotype in db/db mice

**FIGURE 2:**
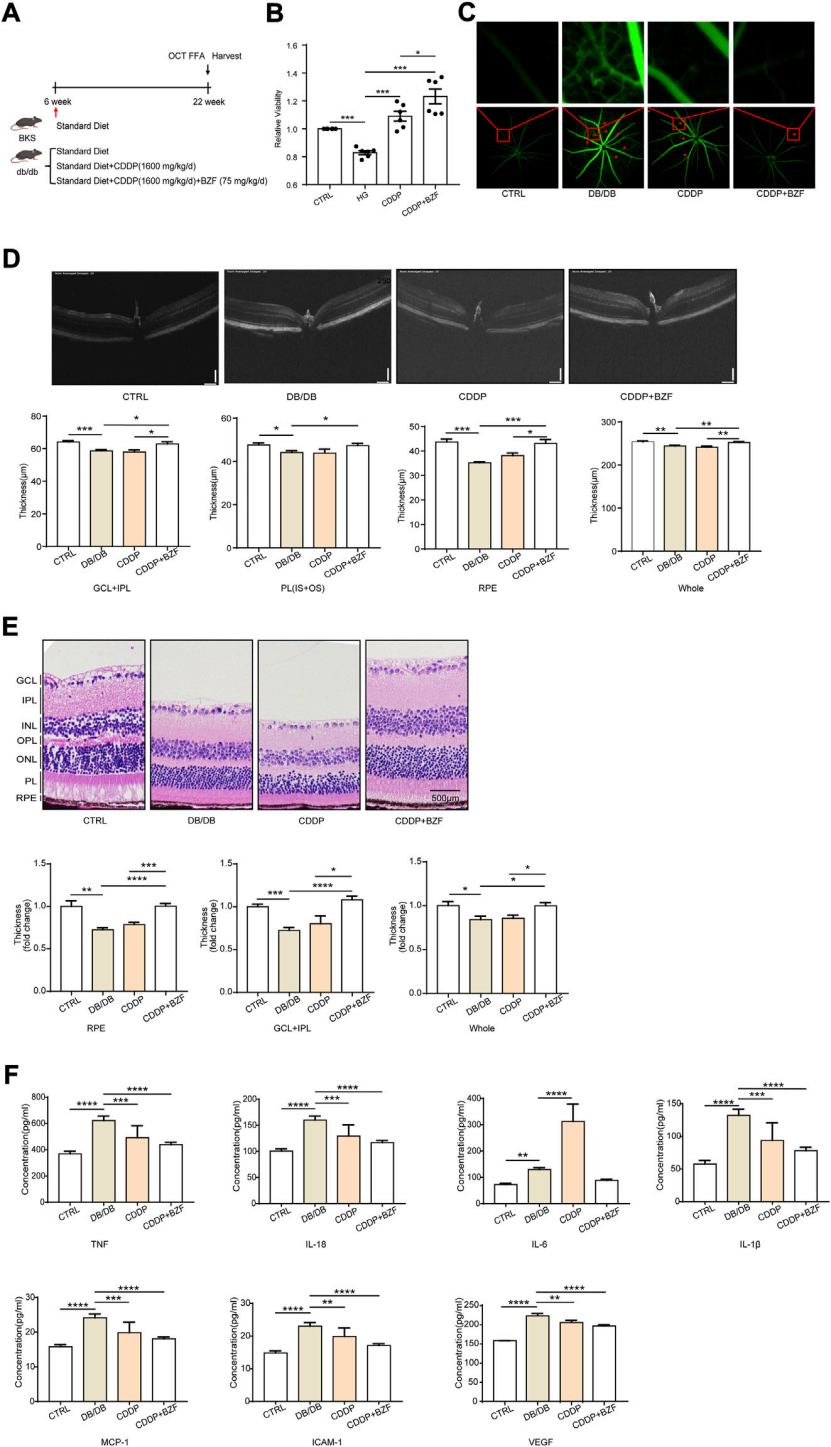
Effects of coadministration of CDDP and BZF on the disease phenotype of DR in db/db mice. **(A)** Graphical experimental protocol for the mice,**(B)** CCK-8 cytotoxicity test, **(C)** FFA fluorescent images of retinal blood vessels, **(D)** OCT images and statistical charts of the fundus oculi of mice, **(E)** H&E-stained sections of the RPE layer in mice (20×), and **(F)** ELISA assays of TNF-α, IL-6, IL-18, IL-1β, MCP-1, ICAM-1 and VEGF in mouse plasma (*n* ≥ 5 in each group). Unpaired *t*-test. *p < 0.05, **p < 0.01, ***p < 0.001, ****p < 0.0001. CTRL: control; HG: high-glucose; GCL + IPL: ganglion cell layer and inner plexiform layer; PL(IS + OS): photoreceptor layer.

To investigate the effects of coadministration of CDDP and BZF on DR, we established a DR disease model using db/db mice (Figure 2A). This model exhibits the pathophysiological characteristics of DR, such as retinal vascular leakage and retinal thinning. Before treating the mice with the drugs, we performed cytotoxicity tests to ensure that the drugs were not harmful to the animals. As expected, the CCK-8 cytotoxicity test showed that coadministration prevented harm to retinal cells (Figure 2B). We treated the mice for 16 weeks with the drugs, and then we measured the retinal vascular leakage by FFA. Fluorescent images of the retinal blood vessels showed that the retinal permeability increased in the DR model group and was rescued in all the treatment groups, which suggested that either coadministration or CDDP monotherapy relieved DR (Figure 2C). We observed the effect of the drugs on retinal tissue thickness using OCT to assess the change in visual acuity. The OCT images showed that the total retinal thickness of the DR model mice decreased dramatically. Some distinguishable tissue layers in the model group were also reduced to different degrees, which included the ganglion cell layer and inner plexiform layer (GCL + IPL), photoreceptor layer (PL), and retinal pigment epithelium (RPE) layers (Figure 2D). Coadministration of CDDP and BZF significantly increased the thickness of all these layers; however, CDDP monotherapy only increased the thickness of the RPE layer. These results show that the combination of drugs increased retinal thickness more effectively than CDDP monotherapy. We used H&E staining to validate the effect of the drugs on the histology of the retina. Consistent with the OCT results, the staining also showed that coadministration of the drugs increased retinal thickness more than CDDP monotherapy (Figure 2E). In particular, the increase in the RPE layer thickness was significantly greater in the coadministration group than in the CDDP treatment group. We also examined the effect of drug coadministration on global inflammation. Serum ELISA experiments showed that BZF reduced the levels of several major proinflammatory factors further, including tumor necrosis factor alpha (TNF-α), interleukin (IL)-6, IL-18, IL-1β, monocyte chemoattractant protein (MCP)-1, intercellular cell adhesion molecule (ICAM)-1 and vascular endothelial growth factor (VEGF) (Figure 2F). In summary, coadministration of CDDP and BZF improved the disease phenotypes of DR, such as retinal vascular leakage, retinal thickness thinning, and increased inflammation.

### Coadministration of CDDP and BZF inhibited retinal cell apoptosis and had a more significant anti-inflammatory effect than CDDP monotherapy

**FIGURE 3:**
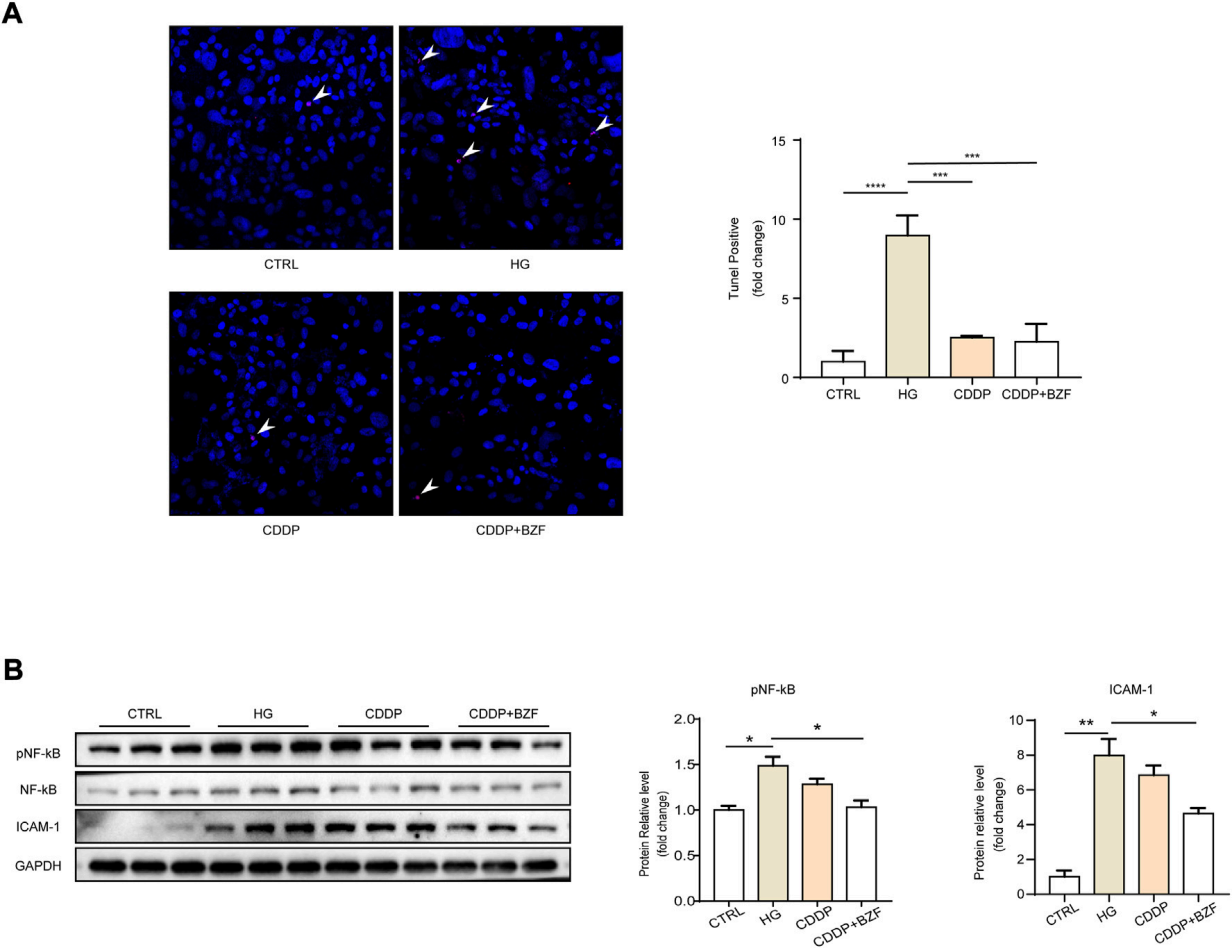
Coadministration of CDDP and BZF inhibited apoptosis and had better anti-inflammatory effects than monotherapy. **(A)** TUNEL positive cell image and its statistical chart (*n* = 5) and **(B)** cell western blot images and statistical charts (*n* = 3). Unpaired *t*-test. *p < 0.05, **p < 0.01, ***p < 0.001, ****p < 0.0001. CTRL: control; HG: high-glucose.

Apoptosis is an important cause of retinal cell loss and consequently retinal thickness reduction. CDDP also inhibits the apoptosis of retinal cells in diabetic rats (Zhang et al., 2018). To investigate whether coadministration of CDDP and BZF affects apoptosis, we established a high glucose induced ARPE-19 cell model that simulated RPE cells in the DR state. We verified the effect of the drugs on the apoptosis of retinal cells induced by high glucose. A TUNEL fluorescence staining experiment showed that the high glucose increased the number of apoptotic cells and both CDDP monotherapy and coadministration of CDDP and BZF inhibited apoptosis (Figure 3A). Inflammation is an important phenotype of DR. Western blotting also showed that CDDP and BZF reduced the protein level of ICAM-1 and the phosphorylation level of its upstream proinflammatory transcription factor, nuclear factor kappa B (NF-κB) subunit p65 (Figure 3B). These results demonstrated that the coadministration group exhibited a stronger anti-inflammatory effect.

### Coadministration of CDDP and BZF had effects against oxidative stress

**FIGURE 4:**
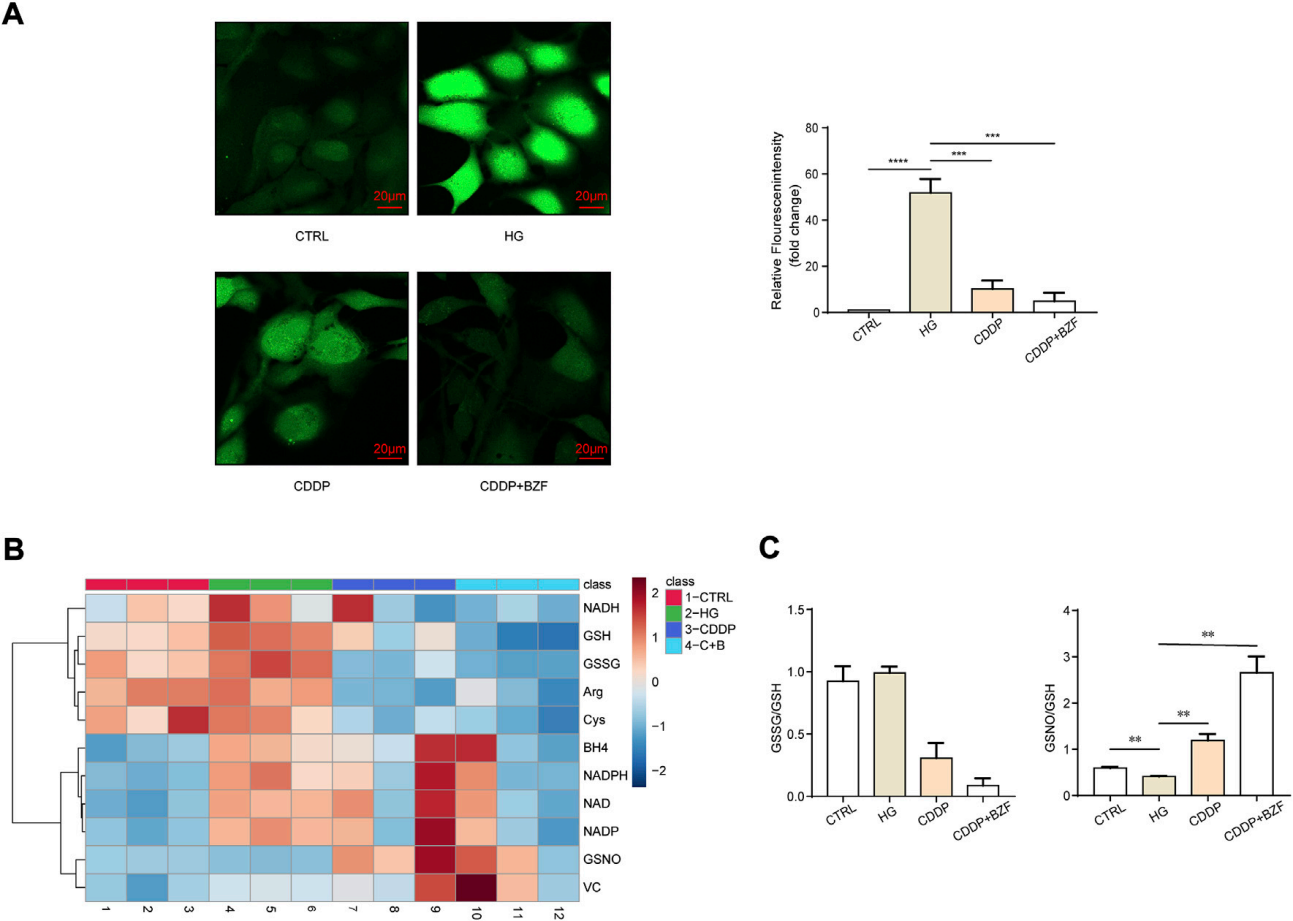
Coadministration of CDDP and BZF had antioxidative stress effects. **(A)** ROS levels in ARPE-19 cells (*n* = 5). **(B)**, **(C)** Metabonomic analysis of ARPE-19 cells (*n* = 3). Unpaired *t*-test. **p < 0.01, ***p < 0.001, ****p < 0.0001. CTRL: control; HG: high-glucose.

Oxidative stress is a main mechanism for both inflammation and apoptosis. First, we observed the effect of the drugs on the oxidative stress level of the retinal epithelium. The ROS level of ARPE-19 was measured by a ROS assay kit. Cell fluorescence images showed that high glucose drastically increased the level of ROS, and both CDDP monotherapy and coadministration with BZF reduced ROS. Moreover, the reduction in the coadministration group was greater (Figure 4A). To investigate the mechanism of the antioxidant effect of the drug further, we examined the small-molecule metabolites associated with the redox state using targeted metabolomics. The heat map showed that both high glucose and drug treatments caused a significant change in redox-related metabolites (Figure 4B). Specifically, coadministration of CDDP and BZF reduced the ratio of oxidized glutathione to reduced glutathione (GSSG/GSH) further than CDDP monotherapy. In addition, coadministration of CDDP and BZF dramatically increased the ratio of *S*-nitrosoglutathione to reduced glutathione (GSNO/GSH), which suggested an increase in the availability of NO (Figure 4C).

## Discussion

The number of patients with diabetes is increasing globally, with an average growth rate of 51%. Approximately 35.5% of people with diabetes develop complications, the majority of which are microvascular complications (Hammes, 2018). As diabetes progresses, DR becomes a more dominant complication. DR is characterized by high morbidity, rapid progression, and a low consultation rate. Most treatments for DR are aimed at the later stages of NPDR or PDR, and the main strategies include anti-VEGF drug injections, total retinal laser photocoagulation, and vitrectomy. There is a lack of effective pharmacological interventions targeting the early stages of DR. CDDP is a traditional Chinese compound medicine used for treating cardiovascular disease and has been approved by the American FDA in phase II clinical trials. Fibrates are agonists of PPARs, and they lower triglycerides and increase insulin sensitivity. Clinical trials have shown that both CDDP and fibrates benefit DR patients (de Moraes and Layton, 2016), (Bogdanov et al., 2015), (Huang et al., 2017), (Lian et al., 2015), but the mechanism is unknown.

The etiology of DR is mainly a series of altered pathophysiological states caused by elevated blood glucose, including hemodynamic abnormalities, local inflammation, and oxidative stress. Both salvinorin and saponin, two of the main components of CDDP, have anti-inflammatory and antioxidant effects (Luo et al., 2015). In addition, BZF, a fibrate drug, significantly inhibited TNF-α-induced expression of MCP-1, ICAM-1 and VCAM-1, and suppressed NF-κB activation in human retinal microvascular endothelial cells (Wang et al., 2021). These pharmacological properties provide the rationale for the mechanism by which CDDP and BZF mitigate DR. Disease proteins are not scattered randomly in the interactome, but tend to interact with each other, forming one or several connected subgraphs called the disease module (Menche et al., 2015). In our work, we created an extremely reliable module for DR by using the LCC approach. Next, based on this DR module, we evaluated the efficacy of BZF and CDDP for DR from the network perspective, and we achieved satisfactory results using network proximity and correlation. We evaluated the network-based proximity of BZF and CDDP using the method proposed by Barabasi et al. (Menche et al., 2015), which may overcome the shortcomings of having a small number of drug targets for a relatively large disease module (Cheng et al., 2019). Our network pharmacology analysis revealed that the targets of CDDP and BZF are both in the disease module, but are in separate neighborhoods in the background network, suggesting that combining the drugs may exert synergistic effects. Therefore, we investigated the protective effect and mechanism of the combination of CDDP and BZF on DR.

We constructed a DR model using db/db mice. After 16 weeks, mice in the db/db group spontaneously developed DR symptoms, such as retinal microvascular leakage, retinal thinning, and elevated serum inflammatory factor levels. We did not observe pathological phenotypes, including retinal macular edema, suggesting that the mice were in the early stages of NPDR. Retinal microvascular leakage is the most representative disease phenotype of DR. FFA images showed that BZF enhanced the ability of CDDP to reduce vascular leakage points, thereby demonstrating that the drug combination provided better DR protection. The thickness of the retina is closely related to visual acuity in DR patients. Previous studies have demonstrated that CDDP increases the thickness of the retina, and thus exerts a protective effect against DR (Liu et al., 2015). The H&E staining and OCT results showed that BZF increased the rescuing effect of CDDP on retinal thickness, which suggested that the drug combination may have a better effect on visual acuity improvement than CDDP monotherapy. The progression of DR is accompanied by inflammation. We used ELISA to detect major proinflammatory factors in mouse serum. TNF-α, IL-6, IL-18, IL-1β, MCP-1, ICAM-1, and VEGF were all increased in the db/db group. CDDP reduced all the proinflammatory factors, except for IL-6, whereas the combination of CDDP and BZF reduced all the proinflammatory factors significantly. These results demonstrated that coadministration of CDDP and BZF exhibited better DR protection than CDDP monotherapy in multiple ways.

We analyzed the mechanism by which BZF produces additional benefits further. The thinning of retinal thickness is probably related to apoptosis, and previous studies have demonstrated the anti-apoptotic effect of CDDP on retinal cells (Zhang et al., 2018). Because the OCT results showed that the most significant effect of CDDP or the drug combination was in the RPE layer, we validated the anti-apoptotic effect of the drug in retinal epithelial cells. Chronic inflammation is extensively involved in the development of DR, the process of which is accompanied by elevated levels of a range of proinflammatory factors, including TNF-α, IL-1β, and MCP-1(Rubsam et al., 2018). Our animal results confirmed that CDDP reduced the levels of most of the proinflammatory factors, thereby reducing inflammation overall. Among the proinflammatory factors that we measured, only the level of IL-6 was upregulated by CDDP. However, the IL-6 levels were eventually downregulated to the basal level by the coadministration of CDDP and BZF, which may reflect the additional protective effect of BZF. The increase of IL-6 is positively correlated with the occurrence and development of DR in rats (Wang et al., 2020). We investigated the effect of drugs on local inflammation further by using cellular models. The coadministration group had a lower level of p65 phosphorylation than the CDDP monotherapy group, which implied that the extra benefits of BZF could be related to NF-κB. Previous work also demonstrated that BZF inhibits the NF-κB pathway, and thus this pathway is involved in preventing apoptosis (Zhong et al., 2011), supporting our hypothesis. Oxidative stress is a main mechanism for both inflammation and apoptosis, and the network pharmacology analysis implied that the mechanism of action of BZF might originate from its antioxidant effect (Figure 1C). Thus, we investigated the effect of the drugs on oxidative stress. The ROS assay showed that CDDP reduced the levels of ROS and coadministration of BZF increased the effect against oxidative stress of CDDP. To elucidate the antioxidant mechanism of the drugs, we examined a variety of small-molecule metabolites associated with redox modulation. GSSG/GSH, nicotinamide adenine dinucleotide (NAD+/NADH), and nicotinamide adenine dinucleotide phosphate (NADP+/NADPH) are the most important redox couples that influence the redox environment of cells, and NAD+/NADH is connected with diabetic cardiomyopathy (Berthiaume et al., 2019), (Chiao et al., 2021). Our study demonstrated that BZF can reduce the ratio of GSSG/GSH further, but cannot affect NAD+/NADH and NADP+/NADPH. BZF has been reported to reduce the GSSG/GSH ratio in brain tissue, thereby exerting a protective antioxidant effect (Dumont et al., 2012). This observation suggested that GSSG/GSH could be a target of BZF in DR. In addition, the ratio of GSSG/GSH is also associated with NF-κB activation (Sen et al., 1997). These clues suggest that the antioxidant effect of BZF may arise from its anti-inflammatory and anti-apoptotic effects. In addition, coadministration caused a significant increase in the GSNO/GSH ratio, which suggested that the availability of NO was elevated by BZF. It has been reported that BZF upregulates the expression of endothelial nitric oxide synthase (Wang et al., 2006). The elevation of NO levels may be a mechanism by which the drug combination achieved better DR protection.

In conclusion, network pharmacology analysis revealed that both CDDP and BZF have potential DR protective effects and the combination of the two drugs act synergistically. Animal experiments demonstrated that the drug combination afforded additional protective effects by resisting vascular leakage, increasing retinal thickness, and reducing inflammation levels. Mechanistically, these protective effects were related to the local anti-inflammatory and redox regulation effects of BZF.

## Data Availability

The original contributions presented in the study are included in the article/Supplementary Material, further inquiries can be directed to the corresponding authors.
